# Inhibition of metabotropic glutamate receptor III facilitates sensitization to alkylating chemotherapeutics in glioblastoma

**DOI:** 10.1038/s41419-021-03937-9

**Published:** 2021-07-21

**Authors:** Julian P. Maier, Vidhya M. Ravi, Jan Kueckelhaus, Simon P. Behringer, Niklas Garrelfs, Paulina Will, Na Sun, Jasmin von Ehr, Jonathan M. Goeldner, Dietmar Pfeifer, Marie Follo, Luciana Hannibal, Axel Karl Walch, Ulrich G. Hofmann, Jürgen Beck, Dieter Henrik Heiland, Oliver Schnell, Kevin Joseph

**Affiliations:** 1 Microenvironment and Immunology Research Laboratory, Medical Center-University of Freiburg, Freiburg, Germany; 2 Department of Neurosurgery, Medical Center-University of Freiburg, Freiburg, Germany; 3 Faculty of Medicine, University of Freiburg, Freiburg, Germany; 4 Translational NeuroOncology Research Group, Medical Center-University of Freiburg, Freiburg, Germany; 5 Neuroelectronic Systems, Medical Center-University of Freiburg, Freiburg, Germany; 6 Research Unit Analytical Pathology, Helmholtz Zentrum München, Neuherberg, Germany; 7 Department of Hematology, Oncology and Stem Cell Transplantation, Medical Center-University of Freiburg, Freiburg, Germany; 8 Department of Medicine I, Medical Center-University of Freiburg, Freiburg, Germany; 9 Laboratory of Clinical Biochemistry and Metabolism, Department of General Pediatrics, Adolescent Medicine and Neonatology, Faculty of Medicine, Medical Center, University of Freiburg, Freiburg, Germany

**Keywords:** Oncogenes, CNS cancer

## Abstract

Glioblastoma (GBM), the most malignant tumor of the central nervous system, is marked by its dynamic response to microenvironmental niches. In particular, this cellular plasticity contributes to the development of an immediate resistance during tumor treatment. Novel insights into the developmental trajectory exhibited by GBM show a strong capability to respond to its microenvironment by clonal selection of specific phenotypes. Using the same mechanisms, malignant GBM do develop intrinsic mechanisms to resist chemotherapeutic treatments. This resistance was reported to be sustained by the paracrine and autocrine glutamate signaling via ionotropic and metabotropic receptors. However, the extent to which glutamatergic signaling modulates the chemoresistance and transcriptional profile of the GBM remains unexplored. In this study we aimed to map the manifold effects of glutamate signaling in GBM as the basis to further discover the regulatory role and interactions of specific receptors, within the GBM microenvironment. Our work provides insights into glutamate release dynamics, representing its importance for GBM growth, viability, and migration. Based on newly published multi-omic datasets, we explored the and characterized the functions of different ionotropic and metabotropic glutamate receptors, of which the metabotropic receptor 3 (GRM3) is highlighted through its modulatory role in maintaining the ability of GBM cells to evade standard alkylating chemotherapeutics. We addressed the clinical relevance of GRM3 receptor expression in GBM and provide a proof of concept where we manipulate intrinsic mechanisms of chemoresistance, driving GBM towards chemo-sensitization through GRM3 receptor inhibition. Finally, we validated our findings in our novel human organotypic section-based tumor model, where GBM growth and proliferation was significantly reduced when GRM3 inhibition was combined with temozolomide application. Our findings present a new picture of how glutamate signaling via mGluR3 interacts with the phenotypical GBM transcriptional programs in light of recently published GBM cell-state discoveries.

## Introduction

There is a rising interest in the metabolic imbalances that are present within the glioblastoma environment, with recent reports highlighting the interface between GBM cells and neural cells within the local microenvironment, driving invasion and proliferation by means of glutamatergic signaling [1, 2]. In addition to glutamatergic inputs from the neuronal environment, GBM has been shown to release glutamate in the nearby environment, interacting with Ca^2+^ permeable AMPA receptors, promoting cellular migration and proliferation through Akt activation [3–6]. The increased level of glutamate in the brain also has clinical relevance, with patients suffering from GBM showing increased cortical levels of glutamate, claimed to be responsible for seizure and excitotoxicity in cells close to the tumor site [7]. In healthy neuronal cells, the mGLuR’s have been shown to control the postsynaptic neuronal response to glutamate, modulating ionotropic glutamate receptor activity, leading to cellular proliferation, growth, migration, survival, and calcium-mediated cellular homeostasis [8, 9]. Besides the reported significance of ionotropic glutamate receptors, the role of metabotropic glutamate receptors (mGluR’s) remains a topic of interest in GBM.

In this work, we demonstrate that glutamate uptake/release is necessary for GBM survival, with glutamate levels being dynamically modulated by cellular stress, validated within a spatial multi-omic dataset. We identified the expression of both ionotropic and metabotropic receptors from a GBM development context and characterized the role each plays in modulating cellular kinetics and proliferation. This enabled us to identify and further characterize the expression of metabotropic glutamate receptor 3 (*GRM3*) in clinical samples, presenting evidence that GRM3 expression was significantly increased and of potential interest in recurrent GBM patients. We report that inhibition of mGluR3 at a non-toxic concentration of 60–100 nM was enough to drive chemotherapeutic resistant GBM to a transcriptional state that resulted in reduced network forming characteristics, which enabled targeted action by means of temozolomide. These results were further validated in our previously published human organotypic section-based tumor model, where tumor growth and proliferation were observed to be significantly reduced when mGluR3 inhibition was carried out in conjunction with temozolomide administration.

## Materials and methods

### Ethical approval

The local ethics committee of the University of Freiburg approved data evaluation, imaging procedures and experimental design (protocol 1000020/09 and 472/15_160880). All methods were carried out in accordance with the approved guidelines. Written informed consent was obtained from all patients. The studies were approved by an institutional review board. Further information and requests for resources, raw data and reagents should be directed and will be fulfilled either by K. Joseph, kevin.joseph@uniklinik-freiburg.de or D.H. Heiland, dieter.henrik.heiland@uniklinik-freiburg.de.

### Cell culture

Three glioblastoma cell lines, CLGSC, CL168, and CL233, were purified from surgical specimens with the informed consent of patients as described previously by our research group [10]. The obtained cell suspensions were then cultured in Minimum Essential Medium (MEM) culture medium (21090022, Thermo Fisher Scientific Inc., Waltham, MA, USA) supplemented with 10% Fetal-calf serum (FCS) (P30-3306; PAN-Biotech GmbH, Aidenbach, Germany) and 1% Penicillin/Streptomycin (15140122, Thermo Fisher Scientific Inc., Waltham, MA, USA). Cell viability and confluence were monitored regularly (48–72 h) by microscopic observation and the culture medium was changed every 5–7 days.

### Viral transduction by constitutive reporter lentiviral vectors

For live imaging and whole-cell tracking, primary cultured glioblastoma cells were transduced with lentiviral particles (rLV.EF1.ZsGreen1-9 and rLV.EF1.mCherry-9 0037VCT, 0038VCT Takara Bio, Kusatsu, Japan) as previously described [11–13]. Briefly, 5 × 10^5^ cells were seeded on a round petri dish. Viral particle quantity was calculated according to the manufacturer’s instructions and the transduction mix was prepared by adding the required volume of thawed viral particles and Polybrene^®^ (10 µg/ml, TR-1003-G, Merck KGaA, Darmstadt, Germany). Cells were then incubated overnight in the transduction mix at 37 °C, 5% CO_2_. Efficacy and quality of transduction was microscopically evaluated after 48 h.

### Glutamate monitoring

Extra- and intracellular glutamate concentrations were determined by using the Glutamate Assay Kit (ab83389, Abcam, Cambridge, UK or MAK004, Sigma-Aldrich, St. Louis, MO, USA) according to the manufacturer’s instructions. For extracellular glutamate concentrations, 50 µl from either cell culture or human organotypic slice cultures was used. Samples were snap-frozen in liquid nitrogen or directly analyzed according to the manufacturer’s protocol. For intracellular glutamate measurements, a dounce glass potter homogenizer was used for mechanical lysis of the cells. As both assays are based on a colorimetric detection method, the amount of glutamate was quantified by measuring the absorbance at 450 nm using a microplate reader (TECAN infinite M200, Zurich, Switzerland). Glutamate concentrations were calculated according to the manufacturer’s calculation guide and stated in µM. For every experiment a glutamate standard curve and background measurements of culture medium and control groups were calculated and taken into account for individual glutamate calculations.

$$\begin{array}{l}Sa =\left(\frac{Corrected \; absorbance-(y-intercept)}{Slope}\right)\\Glutamate\;Concentration=\left(\frac{Sa}{Sv}\right)\ast D\end{array}$$

Sa = Amount of sample (nmol) from standard curve

Sv = Volume of sample (µL) added into the well

D = Sample dilution factor.

### Mass spectroscopy (LC-MS)

Materials. All materials utilized for the targeted analysis of neurotransmitters in tissue biopsies are listed in Table 1.

**Table 1:** List of reagents, supplier and product numbers.

Reagent	Supplier	Product number
D-Serine	Sigma	S4250-5G
L-Serine	Sigma	S4500-1G
Dopamine	Sigma	H8502-5G
Dopamine-D4 hydrochloride solution	Sigma	D-072-1ML
Serotonine hydrochloride	Sigma	H9523-100MG
GABA, γ-amino butyric acid	Sigma	A2129-10G
L-Glutamate	Sigma	49621-250 G
Glycine hydrochloride	Sigma	G2879-100G
Glycine-1-^13^C,^15^N	Sigma	299340-100MG
L-2-Aminoadipic acid	Sigma	A7275-250MG
Tryptophan	Sigma	T8941-25G
Isoprenaline	Sigma	I5627-5G
Water, CHROMASOLV™ LC-MS	Honeywell Research Chemicals	39253-1 L
Methanol, CHROMASOLV™ LC-MS Ultra, tested for UHPLC-MS	Honeywell Research Chemicals	14262-1 L
AC 3 AQ column 1.0 ×150 mm	HiChrom	ACE-116-1501

## Methods

### LC-MS/MS method

The separation and detection of metabolites was performed according to the overall strategy of Xu et al [1], with modifications. Metabolites were separated on a AC 3 AQ column, 1.0 × 150 mm (HiChrom) using a gradient of solvents A (0.1% formic acid in water) and B (0.1% formic acid in MeOH) over 10 min as follows: 0–0.5 min 15% B, 0.5–5 min 85% B, 5–6 min 85% B, 6–6.20 min 15% B, 6.21–10 min 15% B. The flow rate was set to 0.050 mL/min connected to a Nexera X2 LC system (Shimadzu). Metabolites were detected on a Sciex 6500+ ESI-tripleQ MS/MS on low mass mode (0–1000 Da), with curtain gas (CUR) at 40, collision gas (CAD) at 10, Ion spray voltage (IS) at 5000 Volts, temperature (TEM) at 400 ^o^C, ion source gas 1 (GS1) at 40 and ion source gas 2 (GS2) at 30. Each metabolite was optimized individually using chromatographic solvent conditions and exhibited optimal ionization in the positive mode (Table 2).

**Table 2:** Mass transitions and optimized mass spectrometry parameters for the neurotransmitters and internal standards in this study.

Q1	Q3	Dwelling time (ms)	Metabolite	DP	EP	CE	CXP
177,1	160,2	20	Serotonine (5-HT)	38	10	10	7
148,2	84,1	20	Glutamate (Glu)	41	10	24	15
104,2	87,1	20	GABA (g-aminobutyric acid)	26	14	15	16
154,1	137,2	20	Dopamine (DA)	37	10	13	8
205,2	188	20	Tryptophan	40	4	14	12
182,2	136,1	20	Tyrosine	52	8	25	7
212	152,2	20	Isoprenaline	46	8	20	9
76,1	30	20	Glycine (Gly)	6	7,6	19	14
158,1	141,2	20	D4-Dopamine	37	10	13	8
106,1	60	20	Serine (Ser)	6	10,5	15,5	7
78,1	31	20	^13^C, ^15^N-Gly	6	7,6	19	14
105,048	59,1	20	Choline	76	10	39	8
162,055	98,1	20	L-2-AAA	56	10	21	10

### Calibrators and internal standards

All metabolites were quantified with respect to a calibration curve and selected internal standards. The dynamic range of calibrators was 0–5 µM for Glutamate, Serine, dopamine, serotonin and GABA, and 0–100 µM for Glycine, Tryptophan, Tyrosine, Choline and 2-L-Aminoadipic acid. D4- dopamine was utilized as internal standard for the quantification of dopamine. Glycine-1-^13^C,^15^N was utilized as internal standard for the quantification of Glycine. All other metabolites were normalized by isoprenaline as the internal standard, as described in a published method validated according to FDA guidelines [14]. The internal standard mix contained D4-dopamine (5 µM), Glycine-1-^13^C,^15^N (50 µM) and isoprenaline (50 µM).

### Sample handling

#### Tissue biopsies

Flash-Frozen tissue biopsies were stored at −80 ^o^C. On the day of sample preparation, the frozen tissue was weighed quickly by placing it on a small piece of aluminum foil. The tissue was thawed on ice and resuspended with ice-cold lysis buffer (10 mL PBS supplemented with 100 µL protease inhibitor cocktail (P8340-5ML, Sigma-Aldrich, St. Louis, MO, USA)) to a ratio of 100 mg tissue per mL of lysis buffer. The tissue was homogenized on ice with a tissue disruptor (47747-370, VWR, Radnor, PA, US) until the mixture was fully homogeneous. An aliquot of this crude extract was diluted 1:10 with lysis buffer and utilized for metabolomics analysis and protein quantification by the bicinchoninic acid assay (Pierce BCA Protein Assay Kit (23225), Thermofisher Scientific, USA). The remaining undiluted tissue extract was frozen quickly in dry-ice and stored at −80 ^o^C.

#### Sample preparation

Neurotransmitters were extracted according to a fast acidic methanol extraction procedure previously described for polar metabolite extraction [15]. Briefly, 20 µL of calibrator or biological sample of interest was mixed with 20 µL of internal standard mix and 20 µL of 0.1% formic acid in H_2_O. Metabolites were extracted by addition of 100 µL of 0.1% formic acid in MeOH. Samples were vortexed, then centrifuged at 9447 × *g* at room temperature for 10 min and the supernatants were transferred into HPLC vials.

#### Data analysis

Quantification of metabolites was carried out with Analyst® 1.6.3 software, 2015 AB Sciex.

### Cell proliferation ELISA, BrdU (colorimetric)

For the assessment of cell proliferation, the colorimetric BrdU ELISA immunoassay by Roche (11647229001; Roche, Basel, Switzerland) was used according to the manufacturer’s instructions. This method is based on the measurement of BrdU incorporation during DNA-synthesis in replicating cells. For all Cell Proliferation ELISA, BrdU (colorimetric) experiments, 5 × 10^3^ cells were seeded per well (100 µl) on 96-well culture plates (655180; Greiner Bio-One International GmbH, Kremsmünster, Austria). For every experiment, triplets of all different conditions and at least one untreated control group were set up. Cells were incubated for 48 h at 37 °C, 5% CO_2_ to ensure satisfying cell viability and confluency before the treatment solutions were administered. Treatment periods were terminated by replacing the treatment solutions with MEM culture medium, followed by 6 h BrdU incorporation with BrdU labeling solution according to the manufacturer’s instructions. Colorimetric measurements were performed after the addition of 25 µl 1 M H_2_SO_4_ (stop solution) to each well and 1 min incubation on a shaker at 300 rpm. A microplate reader (TECAN infinite M200, Zurich, Switzerland) measured the absorbance at 450 nm in reference to wavelength 690 nm.

### Cell proliferation and cytotoxicity assay (WST-8)

Cell Proliferation and cytotoxicity was assessed by using the Cell Counting Kit 8 (WST-8) (ab228554, Abcam, Cambridge, UK). This method is based on the colorimetric change of WST-8/CCK tetrazolium salt when reduced to an orange formazan dye by cellular dehydrogenases. Briefly, cells were seeded at a density of 5 × 10^3^ cells per well (100 µl) on 96-well plates (655180, Greiner Bio-One International GmbH, Kremsmünster, Austria) and incubated for 48 h at 37 °C, 5% CO_2_ to ensure satisfying cell viability and confluency. Cells were then treated for usually 24 h at 37 °C, 5% CO_2_, before the WST-8 assay was performed. After termination of the treatment period, all treatment solutions were discarded and replaced. According to the manufacturer’s instructions, 10 µl WST-8-solution was added to each well, followed by 4 h incubation at 37°C, 5% CO_2_, protected from light. Absorbance was read at a wavelength of 460 nm using a microplate reader (TECAN infinite M200, Zurich, Switzerland).

### IncuCyte S3 live-cell analysis

The IncuCyte S3 Live-Cell Analysis System (Essen BioScience Inc., Sartorius AG, Göttingen, Germany) was used for image-based analyses and whole cell-tracking of glioblastoma cell lines under different treatment conditions in a time-dependent manner. By using ZsGreen and mCherry transfected GBM cell lines, it was possible to investigate individual cell proliferation by cell count statistics, as well as profiling cellular activity and migration. Therefore, 1.5 × 10^3^ cells per well were seeded on 96-well plates (655180; Greiner Bio-One International GmbH, Kremsmünster, Austria) and incubated for 48 h at 37 °C, 5% CO_2_, to ensure satisfying cell viability and confluency. Thereafter, cells were treated with different treatment solutions and incubated at 37 °C, 5% CO_2_. Fluorescence images were acquired in various time-intervals (10 min–3 h) for a total time period of up to 3 days. Optical modules were set for up to two channels, green (acquisition time 100 ms) and red (acquisition time 100 ms), using the 10x objective. The obtained data were processed with the device-specific software (IncuCyte 2019B) to precisely determine individual cell counts. Additional migration and activity tracking were performed using CellTracker software and post-processed by celltracer R software package.

### Kinetic apoptosis kit

Abcam’s Kinetic Apoptosis Kit (Microscopy) (AB129817, Abcam, Cambridge, UK) was used for the visual assessment of early and late apoptotic events. The assay is based on pSIVA™, an Annexin XII-based polarity sensitive probe which is conjugated to IANBD, a polarity sensitive dye that fluoresces only when bound to phosphatidyl-serine in the non-polar environment of the membrane lipid bilayer of the cells. The pSIVA-IANBD *(green)* based assay was used in combination with propidium iodide (PI, *red*) to distinguish between non-apoptotic (pSIVA-IANBD negative/PI negative), early apoptotic (pSIVA-IANBD positive/PI negative), and late apoptotic or necrotic cells (pSIVA-IANBD positive/PI positive), respectively. According to the manufacturer’s instructions, 20 µl of pSIVA-IANBD and 10 µl of PI per 1.5 ml of culture medium were used. Microscopic imaging was performed by using the IncuCyte S3 Live-Cell Analysis System as described in the previous section.

### Cellular kinetics analysis

The images obtained using the IncuCyte S3 Live-Cell Analysis were loaded into the image software CellTracker [16] and underwent vignetting correction and automatic alignment. Subsequently cells were tracked semi-automatically. This option requires the user to manually tag all cells that are supposed to be tracked in the first image. The software than proceeds with the tagged cells and tracks them over all images. The ‘Timepoint data’ was kept and subsequently processed using the R-Software package “celltracer” (https://github.com/kueckelj/celltracer). The celltracer-object was set up with the function celltracer::initiateCTO(). Only cells that were tracked from the first till at least the second last image were included in downstream analysis. Cells that did not meet this quality requirement were discarded. A total of 9867 cells were analyzed. Figures were plotted using the following functions:

Figures 4e, 5c—celltracer::plotAllTracks

**Fig. 1:**
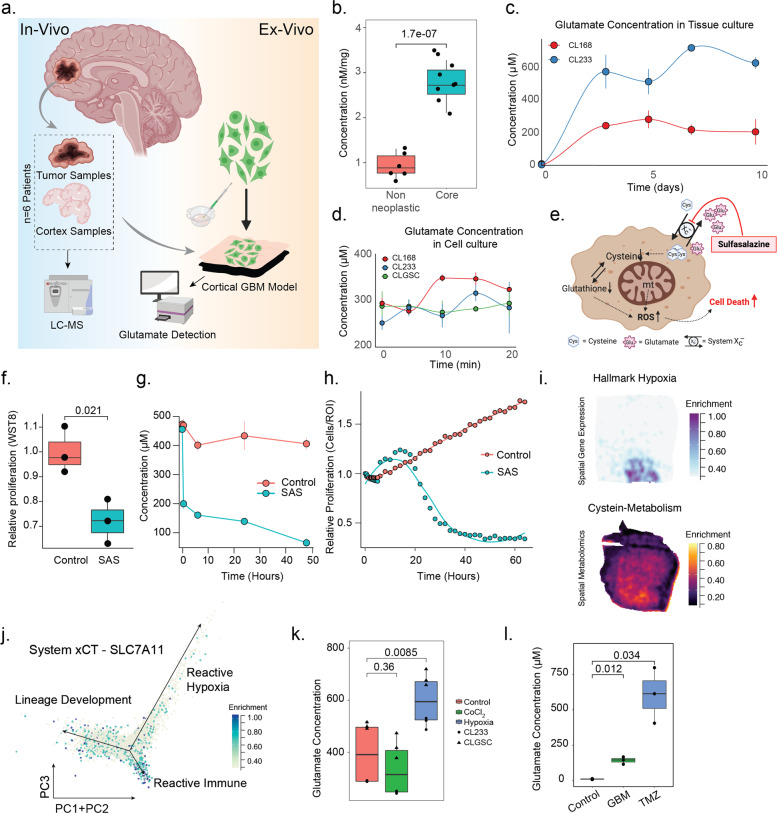
Glutamate dynamics in GBM. **a** Workflow of the experimental paradigm used to study glutamate concentration within a GBM microenvironment. **b** Mass Spectroscopy based measurements of glutamate concentration from tissue samples sourced from the tumor core vs non infiltrated tissue show a significantly higher concentration of glutamate in the core (*n* = 6 samples, *p* < 0.001, *t* test). **c** Healthy cortical tissue was inoculated with GBM cells and cultured over a period of 9 days. The growth medium was collected over the culture period and the glutamate concentration was quantified over a time period of 9 days. **d** Glutamate concentration in cell culture reaches a plateau within 1 min of medium swap. The maximum concentration is reached within 10 mins, which then maintained over the measurement period. **e** Mechanism of action of sulfasalazine, where the inhibition of the Glutamate-Cysteine transporter leads to an increase in ROS within the cells, leading to cell death. **f** Sulfasalazine treatment has a significant impact on Proliferation, as measured by WST-8 proliferation assay (*n* = 3, *p* = 0.021). **g** Inhibition of the Glutamate/Cysteine symporter by means of Sulfazalazine (500 μM) results in a significant reduction in the extracellular glutamate concentration over 48 h. **h** Relative proliferation of cells treated with SAS shows a significant decrease in comparison to control cells. **i** Spatially resolved expression profile of Hypoxic regions shows an overlap with spatially resolved metabolomics of cysteine metabolism. **j** Expression of SLC7A11 within GBM samples show an enrichment within reactive hypoxia subtype of GBM. **k** Exposure of GBM to hypoxic conditions result in a significant increase in glutamate concentration (*n* = 3, *p* = 0.0085). This increase in glutamate concentration was not observed in artificial hypoxia, by CoCl_2_ (*n* = 3, *p* = 0.36). **l** Glutamate concentration measurements from tissue culture experiments where cortical sections were inoculated with GBM shows a significant increase in comparison to control sections (*n* = 3, *p* = 0.012). When the sections were further treated with TMZ, a significant increase in glutamate concentration was seen with respect to control sections (*n* = 3, *p* = 0.034). All box plots represent median, with hinges representing 25th and 75th percentile and whiskers representing 1.5x Interquartile range.

**Fig. 2:**
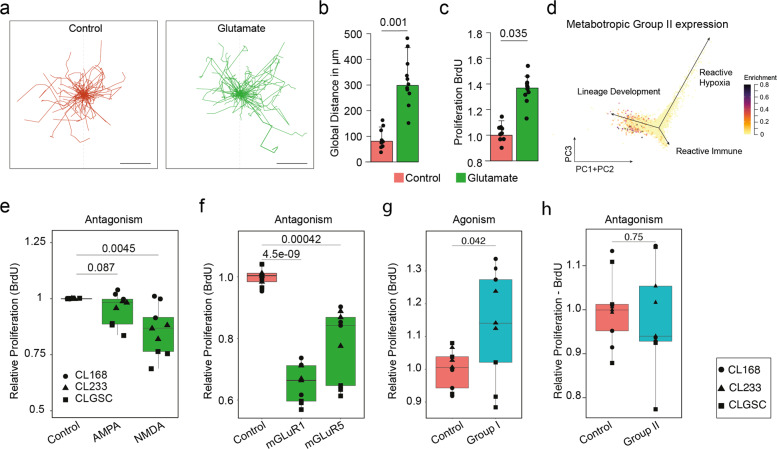
Glutamate receptors and their function in GBM. **a** Glutamate stimulation shows that there is a significant increase in the movement in comparison to unstimulated controls (*n* = 24 cells each). Scale bar = 50 μm. **b** Quantification of the global distance traveled shows that there is a significant increase in the global distance traveled by GBM cells that are stimulated with glutamate vs unstimulated controls (*n* = 24 each, *p* = 0.001). **d** Expression of Metabotropic Group II receptors was localized to the lineage subtype of GBM cells, with minimal expression in the reactive subtypes. **e** Antagonism of AMPA and NMDA receptors revealed that only the inhibition of NMDA receptors resulted in a decrease in cell proliferation (*n* = 9, *p* = 0.0045). **f** Antagonism of Group I metabotropic receptors (mGLuR1 and mGLuR5) resulted in a significant reduction in cell proliferation (*n* = 9, *p* < 0.001). **g** Agonism of Group I receptors significantly increased the proliferation of GBM (*n* = 9, *p* < 0.05). **h** Agonism of Group II metabotropic receptors show no change in proliferation (*n* = 9, *p* > 0.05). All box plots represent median, with hinges representing 25th and 75th percentile and whiskers representing 1.5x Interquartile range.

Supplementary Figs. 4d, 5a—celltracer::plotVelocityHeatmap

Supplementary Figs. 4f, 5b—celltracer::plotBoxplot

Supplementary Figs. 5c—celltracer::plotRidgeplot

### Human organotypic slice model

#### Pre-sectioning preparation

To avoid any contamination of the tissue during the cortical sectioning, a sterile working field was set up for the whole sectioning procedure. All tools required for the procedure were sterilized following clinical hygiene protocols and placed within reach of the experimenter to prevent any delays. A vibratome (VT1200; Leica Biosystems) was used to prepare the sections. To maintain the best-possible tissue environment, the sectioning chamber was filled with ice-cold preparation medium (see Supplementary Table 3. *Preparation Medium Human Organotypic Slice Model*), saturated with carbogen (95% O_2_ and 5% CO_2_). Millicell® cell culture inserts (PICM0RG50, Merck KGaA, Darmstadt, Germany) were placed in each well of six-well culture plates and filled with 1 ml of growth medium.

#### Preparation of human organotypic brain slices

All tissue blocks were directly transferred from the OR to the laboratory and submerged in preparation medium. Subsequently, all visibly damaged areas of the tissue were dissected away. After mounting the processed tissue block on the vibratome, 300-µm-thick sections were obtained at 0.12 mm/s and were incubated in cold preparation medium (4 °C) for 10 min before plating on Millicell® Cell Culture Inserts. The entire growth medium was replaced 24 h post-plating and every 48 h thereafter. From each donor sample, three to four sections were freshly fixed in 4% PFA post sectioning and used as control specimens. At the terminal time point, sections were fixed in 4% PFA for further immunofluorescence-based staining.

#### Tumor injection onto tissue cultures

For monitoring the tumor invasion and proliferation profile in the brain sections, ZsGreen transfected glioblastoma cell lines (CLGSC, CL233) were used. Therefore, the ZsGreen transfected GBM cell lines were trypsinized and counted by using a hemocytometer. Post-trypsinization, 1 × 10^6^ cells were harvested, centrifuged (200 × *g*; RT; 5 min) and resuspended in 50 µl PBS to a final concentration of 20.000 cells/µl. The obtained cell suspension was immediately used for tumor injection onto the brain section. For the injection, a 10 µl Hamilton syringe (80330; Hamilton Company, Bonaduz, Switzerland) was used to manually inject ~1 µl of the GBM cell suspension into the white-gray matter interface in the sections. Sections with injected GBM tumor cells were incubated at 37 °C, 5% CO_2_, and fresh culture medium was added every 24–48 h. Tumor proliferation was monitored by imaging at day 0, 2 post-injection (DPI) and day 2, 4, and 6 post-treatment (DPT) by using an inverted fluorescence microscope (Zeiss, Observer D.1).

#### Immunofluorescence (IF)

Sections were washed three times with PBS for 5 min each and fixed in 20% methanol solution for 5 min at room temperature. After another three washing cycles with PBS, the sections were incubated overnight at 4 °C in 1% Triton solution. The sections were then washed twice with PBS and subsequently blocked with a Triton-BSA (20% BSA + 0.1% Triton X-100) blocking solution for 4 h at room temperature. After another three PBS wash cycles, the primary antibodies were diluted in a Triton-BSA (1:500, 5% BSA + 0.1% Triton X-100) mix and incubated overnight at 4 °C. All sections were then washed twice with PBS before the sections were incubated with secondary antibodies (1:1000,0.1% Triton+5% BSA) for 2 h at room temperature. Finally, the sections were washed twice and transferred to microscopic slides. For all primary and secondary antibodies, appropriate concentrations were used according to the manufacturer’s instructions as indicated in Supplementary Tables 1, 2. Fluorescence microscopy was performed by using the Fluoview FV10i confocal microscope from Olympus. All measurements and image processing were performed using the company’s software and FIJI [17].

#### Immunohistochemistry (IHC)

For immunohistochemical analysis, 30 samples of 18 patients with available de novo (R0), first recurrence (R1) or paired samples of R0 and R1 tissue samples were selected. All samples were sourced from patients diagnosed with WHO Grade IV Glioblastoma (GBM). Tissue samples were fixed in 4% formaldehyde and paraffin-embedded according to standard procedures as described previously [18]. Immunohistochemistry Application Solutions Kit (Rabbit) (13079 S, Cell Signaling Technology, Danvers, USA) was used for all immunohistochemical staining. Deparaffinization and heat-induced epitope retrieval (HIER) in citrate buffer of 3 µm paraffin-embedded tissue sections were performed according to the manufacturer’s instructions. Sections were incubated overnight at 4 °C in anti-GRM3 antibody (ab188750, 1:250, Abcam, Cambridge, UK). The sections were then incubated in SignalStain Boost Solution and Secondary Antibody Solution (DAB-Solution), followed by counterstaining with Meyer’s haemalaun solution and mounted on glass slides. Imaging was carried out using an Olympus BX40 microscope. GRM3 positive cells of 4-6 optical fields (20x magnification) were counted per slide, and the mean number of GRM3 positive cells per ROI was calculated.

### Statistical analysis of clinical data

For analysis of clinical data, a Cox Regression model was used. Kaplan Meier Survival plots were built by the survival package in R software environment [19]. The ranked plots of IHC GRM3 protein expression and survival were plotted using a custom function implemented in R. Code available on reasonable request.

### RNA extraction and isolation

For Microarray Gene Expression Analysis, all glioblastoma cell lines used in this study were profiled. Biological independent triplets were set up for all conditions, with 200.000 cells resuspended in 5 ml of MEM culture medium and cultured in 25 cm^2^ cell culture flasks at 37 °C, 5% CO_2_. For treatment conditions, cells were treated with freshly prepared treatment solutions according to the manufacturer’s instructions. Based on previous results of cell viability and apoptosis analysis by the Kinetic Apoptosis Kit, cells were treated for 4 h at 37 °C, 5% CO_2_. Thereafter, the supernatant of every sample was transferred to 15 ml falcon tubes to prevent any cell loss in the case of cell-detachment during the treatment period. The cell culture flasks were washed with 2 ml of PBS, which was also collected in 15 ml falcon tubes. Remaining adherent cells were enzymatically detached by 1 ml Trypsin 0,25% for 2–3 min at 37 °C, 5% CO_2_. The enzymatic reaction was stopped by the addition of 3 ml MEM culture medium containing 10% FCS and the cell suspension was transferred to the 15 ml falcon tubes. The collected cell suspensions were centrifuged for 10 min at 3000 × *g* and the supernatant was discarded. Subsequent RNA extraction and isolation was performed by using the PicoPure RNA Isolation Kit (KIT0204,Thermo Fisher Scientific Inc., Waltham, MA, USA) for RNA Extraction according to the manufacturer’s instructions. To avoid any RNase contamination, only new certified nucleic acid-free plasticware, as well as only new, sterile, RNase-free pipette tips and microcentrifuge tubes were used throughout the experiment. To avoid the introduction of RNase, all work surfaces were cleaned with RNase decontamination solution.

### RNA quantification and quality assessment

For the accurate quantification of our RNA sample concentrations, the Invitrogen Qubit RNA BR (broad range) Assay Kit (Q10210; Thermo Fisher Scientific Inc., Waltham, MA, USA) was used according to the manufacturer’s instructions. Concentration differences between samples were equalized by dilution with elution buffer to a consistent RNA concentration (100 ng / 3 µl) of each sample. Additionally, sample quality was controlled by performing the Bioanalyzer RNA 6000 Nano assay with RNA Nano Chips and the 2100 Bioanalyzer instrument according to the manufacturer’s instruction, and subsequent analysis with 2100 Expert Software (Agilent Technologies, Santa Clara, CA, US).

### Reverse transcription and microarray transcriptome profiling

The Clariom^TM^ D Assay, human (902925 Clariom D Pico Assay, human, Thermo Fisher Scientific Inc., Waltham, USA) was used for RNA to cDNA conversion and Microarray Transcriptome Profiling and was carried out using an Affymetrix GS3000 GeneChip System.

### Microarray gene expression analysis

Data from microarray sequencing was background corrected and scaled using the LIMMA package, in the R computing environment. Differential gene expression analysis was carried out using DeSeq2 [20]. Further analysis and plots were generated out using our inhouse VisLab package (heilandd/Vis_Lab1.5: Vis_Lab1.5 (github.com)) Gene expression networks were generated using the clusterprofiler package in R [21].

## Results

### Glutamate enrichment in the Glioblastoma microenvironment

The presence of increased glutamate in and around the vicinity of GBM cells has been described previously [22]. GBM has been shown to make use of glutamate for a variety of metabolic and signaling processes, in conjunction with the local microenvironment [2, 23, 24]. We performed LC-MS based neurotransmitter quantification from paired cortical and tumor samples, which showed a significant difference between the tumor and cortical samples, marked by high concentrations of glutamate in the tumor (*p* < 0.001, *n* = 6), Fig. 1a, b. In order to investigate the dynamics of tumor glutamate release, we used a neocortical slice model where human tissue sections were cultured for 2 days, followed by GBM inoculation and further cultured for an additional 9 days (Workflow, Fig. 1a). Cortical sections without tumor cells showed concentrations of glutamate <10 µM which is consistent with literature for the healthy cortex [22, 25, 26]. Culture medium collected from GBM injected sections showed a significantly increased concentration of released glutamate (470.6 ± 257.6 µM, Fig. 1c). These findings are in agreement with experiments carried out in tumor cell culture, where the glutamate concentration was ~300 µM, throughout the culture period. This glutamate level was found to be rapidly restored to values seen in long term culture, reaching a plateau concentration of 273.7 ± 21.6 µM within <60 s, as has been previously reported in the case of astrocytes, Fig. 1d. These dynamics are confirmed to be due to the large intracellular glutamate stores, which was significantly higher than measured extracellularly (extra- vs. intracellular; 295.2–325.8 µM vs. 599.7–695.0 µM; *p* < 0.0001), Supplementary Fig. 1a. This remarkable variation between cell lines which was observed in our GBM model is less prominent in in vitro cultures, suggesting that the neural microenvironment plays a major role in regulating glutamate homeostasis.

**Fig. 3:**
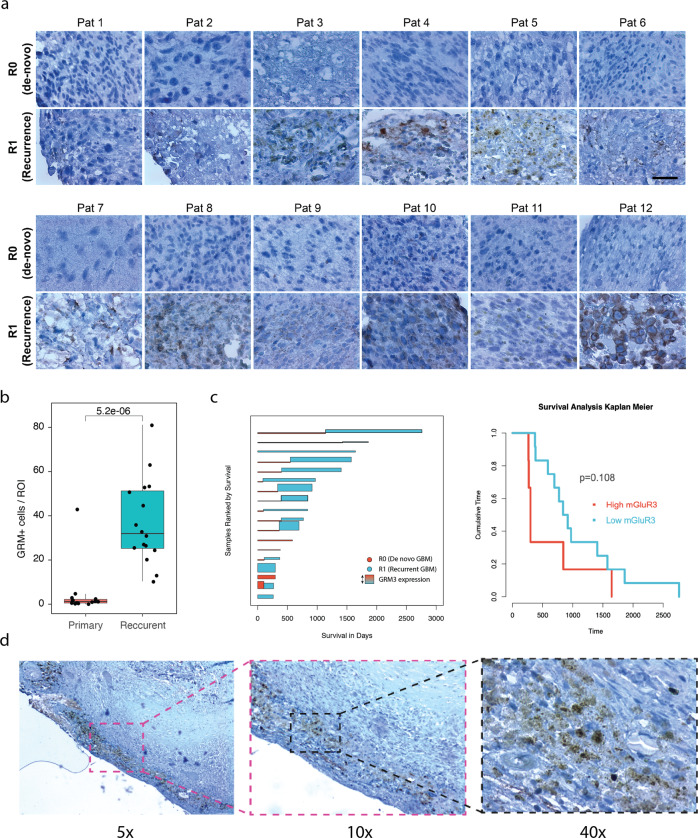
GRM3 expression in GBM patients. **a** Patient derived samples show that there is a significant increase in GRM3^+^ cells in recurrent patients. **b** Quantification of GRM3^+^ cells within the samples show that there is a significant increase in the number of cells in the recurrent samples in comparison to de novo samples (*n* = 18 patients, 12 paired samples). Scale bar is 50 µm. **c** Survival analysis from the patient cohort reveals that there is a trend towards increased patient survival when there are fewer GRM3^+^cells within the tumor. **d** Magnified image of a patient sample containing GRM3^+^ cells. Box plot represents median, with hinges representing 25th and 75th percentile and whiskers representing 1.5x Interquartile range.

These high levels of extracellular glutamate have been reported to be beneficial in the maintenance of tumor cell viability by reduction of reactive oxygen species (ROS, in hypoxic regions), through cysteine-glutamate exchange [24], Fig. 1e. The glutamate antiporter xCT exports intracellular glutamate in conjunction with cysteine import. To validate the role of xCT in glutamate release, we made use of an xCT inhibitor, Sulfasalazine (SAS, 500 µM) [27, 28], which revealed a significant reduction of extracellular glutamate (~70 µM vs. ~400 µM, *p* < 0.00001). In addition, we observed that SAS treatment results in a significant reduction in proliferation (*p* = 0.021, *n* = 3) and increase in apoptosis, Supplementary Figs. 1b-c, 1f–h. These findings suggest that the xCT could play a significant role in cellular viability within hypoxic regions of the tumor. To investigate the spatial metabo-transcriptomic architecture within hypoxic regions, we made use of a recently published dataset, showing a significant enrichment of cysteine metabolism within hypoxic regions [29], Fig. 1i. Gene expression of System xCT (Gene: *SLC7A11*) was pronounced within the reactive subtype of GBM Fig. 1j. Administration of hypoxia (3% O_2_) to primary cell cultures confirmed an increased release of glutamate, not observed in artificial hypoxia (CoCl_2_), Fig. 1k. Further treatment with a common chemotherapeutic (Temodal, TMZ) also revealed a significant increase in extracellular glutamate levels, suggesting that stress induced by toxicity leads to increased glutamate release, Fig. 1l. Therefore, we hypothesize that glutamate release might also play a role as an autocrine stimulator to help mitigate the effects of toxic stress.

### Rapid enrichment of the local micro-environment with cytotoxic levels of glutamate

To further study the effects of autocrine stimulation by secreted glutamate, we estimated the cellular kinetics due to glutamate stimulation, which revealed an increased migratory behavior in stimulated cells (*n* = 100, *p* = 0.001, Fig. 2a, b, Supplementary Fig. 2a–d). Glutamate stimulation also led to increased proliferation (*p* = 0.035, Fig. 2c, Supplementary Fig. 2a). Characterization of the expression of Glutamatergic receptors in publicly available transcriptomic data [29] from GBM revealed that AMPA receptors are restricted to cells within the developmental subtype, whereas NMDA receptors were expressed within all lineage states. Exploration of the Metabotropic glutamate receptors revealed a weak expression of Group III receptors, with Group I and Group II receptors preferentially expressed within the developmental niche, Fig. 2d, Supplementary Fig. 2b. Modulation of ionotropic receptors showed some impact on cell proliferation, in particular, antagonism of the AMPA receptors (NBQX, 50 µM) showed no significant difference in proliferation and migration (*p* = 0.087), whereas antagonism of NMDA receptors (AP5, 50 µM) resulted in significant reduction in cellular proliferation and migration (*p* < 0.01), Fig. 2e. Inhibition of the metabotropic Group I receptors (mGluR1 and mGluR5) results in a significant reduction of cell proliferation (*p* < 0.001), Fig. 2f. Conversely, treatment with an agonist of Group I receptors resulted in a significant increase in cell proliferation, above what is seen with glutamate stimulation alone (*p* = 0.042), Fig. 2g. Inhibition of metabotropic group II/III receptors (LY341495, 60 nM) showed no effect on cell proliferation (*p* = 0.75), Fig. 2h. In order to target the autocrine mechanisms leading to cellular survival and resistance, we aimed to identify a receptor group that was not directly required for cellular viability but rather disables cellular response to toxic stress. Our data suggests that the metabotropic Group II/III are not crucial for cellular survival but might play an important role in evading cellular stress. There has been evidence presented that these receptors play an important role in synaptic plasticity, spine maturation and circuit development [30], all of which have been reported to be crucial for GBM growth in the brain [2]. In addition, there have been previous reports detailing the inhibition of mGLuR3 (Group II receptor) sensitizing GBM cells to chemotherapeutic agents in in vitro models [31].

**Fig. 4:**
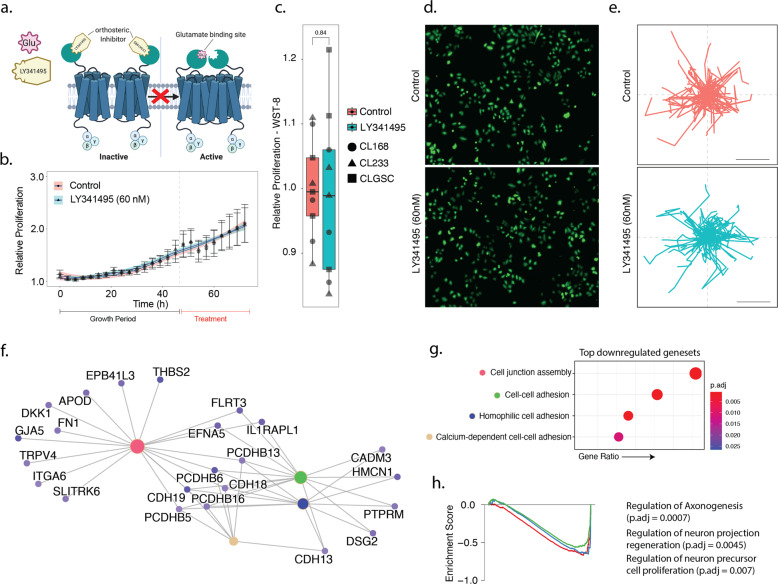
The effect of mono-therapeutic stimulation using LY341495. **a** Mechanism of action of LY341495. LY341495 is a competitive antagonist for the mGluR3 receptor. **b** Live imaging experiments carried out over a 72 h time period shows that there is no difference in proliferation as a result of the treatment with LY341495. **c** Stimulation with LY341495 shows no change in proliferation, measured using WST-8 (*n* = 9, *p* = 0.94). **d** Representative Images showing that there is no change in cell viability as a result of mGLuR3 inhibition by LY341495. **e** Quantification of cellular kinetics. Time-lapse imaging was carried out over 72 h and features pertaining to movement were extracted from *n* = 100 cells per condition. Migration plots from cells that were treated with LY341495 versus unstimulated controls shows no difference in the distance traveled (*n* = 100 each). Scale bar is 50 µm. **f** Transcriptomic analysis shows a significant reduction in expression of genes related to cell junction assembly and cell-cell adhesion. **g** Geneset enrichment analysis shows a downregulation of pathways related to cellular adhesion and tumor formation. **h** Geneset Enrichment Analysis shows a significant downregulation of neural related pathways. All box plots represent median, with hinges representing 25th and 75th percentile and whiskers representing 1.5x Interquartile range.

### mGluR3 receptor expression in GBM

To investigate the role of GRM3 expression in glioblastoma samples, we profiled paired samples obtained from 12 patients, both de novo and recurrent WHO Grade IV Glioblastoma for mGluR3 expression, Fig. 3a. mGluR3 expression was found to be significantly increased in recurrent samples, compared to de novo tumor samples (n = 12, *p* < 0.0001), Fig. 3b. Higher overall survival was seen in patients with low expression of mGluR3, with increased expression observed more frequently in patients suffering from recurrent glioblastoma, negatively correlated with the overall survival, Fig. 3c. All recurrent tumor samples were acquired from patients that underwent a combined radio and chemotherapy protocol (STUPP treatment) [32]. These clinical findings, in combination with our data strengthened the relation between increased mGluR3 expression as a mechanism of chemoresistance. In addition, regional heterogeneity in mGluR3 expression was observed, with mGluR3^+^ cells preferentially observed in the leading-edge zone, Fig. 3d. Highest expression of mGluR3^+^ cells was observed along the leading edge of the glioblastoma, which is consistent with transcriptomic data, with *GRM3* expression predominantly in the neurodevelopmental subtype of GBM, Fig. 2d. Similar results were reported in the GlioVis data portal [33], with mGLuR3 (*GRM3*) expression most pronounced in the infiltrating region of the tumor, Supplementary Fig. 3a.

**Fig. 5:**
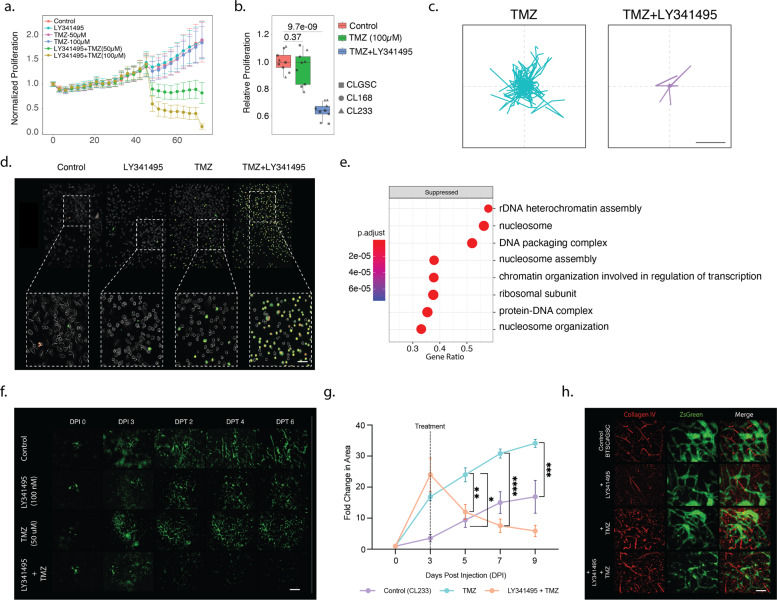
Efficacy of neo-adjuvant therapy with GRM3 inhibition in experimental conditions. With TMZ resistant cell lines (**a**) Quantification of time lapse imaging experiments show that TMZ alone had minimal effects of cellular proliferation. When combined with LY341495, there is a significant reduction in proliferation. The effect is potentiated when the dosage of TMZ is increased to 100 µM. **b** Relative proliferation measured by BrdU shows that there is no change in proliferation as a result of treatment with TMZ (*n* = 3, *p* > 0.05), whereas there is a significant decrease in proliferation when cells were treated with the combined approach (*p* < 0.0001). **c** Quantification of cellular migration shows that there is a significant reduction in cellular migration as a result of the combination therapy. **d** Kinetic apoptosis measurement. Cells in early apoptosis bind to pSIVA, showing green fluorescence. When the cells are necrotic, PI is able to bind to the nuclear membrane, leading to red fluorescence. Kinetic apoptosis assay shows that the combined treatment is the most effective in inducing terminal PI^+^ cells within 4 h of treatment. The cells treated with the combination therapy appear yellow due to the expression of both pSIVA (green) and PI (red). Scale bar is 50 μm. **e** Geneset enrichment analysis from samples treated with TMZ vs. TMZ + LY341495 (**f**) Growth profiles of GBM model sections under different treatment conditions. The combination treatment showed the most effectivity in reducing tumor growth. Scale bar is 250 μm. **g** Quantification of tumor growth over time shows that there is a significant reduction in the tumor growth in the combined treatment strategy. There is no reduction in tumor growth in control sections and TMZ treated (50 µM) sections post treatment, whereas a strong reduction in tumor area is seen in the sections treated with the combination therapy. **h** Immunostainings of collagen within the injected slices show GBM cells growing along blood vessels in control, LY341495 and TMZ treated conditions. When the combination therapy was used, we see a strong decrease in the GBM cell growth. Scale bar is 50 μm. The box plot represents median, with hinges representing 25th and 75th percentile and whiskers representing 1.5x Interquartile range.

### mGluR3 inhibition leads to changes in gene expression, but does not affect survival and cellular kinetics

We induced mGluR3 inhibition by means of LY341495, a highly potent and selective antagonist for Group II metabotropic glutamate receptors, at nanomolar concentrations (60 nM, Fig. 4a). Inhibition of mGluR3 neither affected GBM cell proliferation nor cell viability in cell culture, as well as morphology, Figs. 2h, 4b–d, Supplementary Fig. 4a, b. Quantification of cellular kinetics shows that there is no significant difference in cellular movement and all other measured features, Fig. 4e, Supplementary Fig. 4c–f. To gain insights into the transcriptional effects of inhibition, we performed microarray-based transcriptomic analysis. Top 100 most downregulated genes were related to pathways involving cell junction assembly and cell-cell adhesion, Fig. 4f. GSEA analysis shows that there was a significant downregulation of pathways related to neurite growth (GO: *regulation of neuron projection regeneration*, *negative regulation of nervous system development*), Fig. 4f, h. Recent reports have pointed to the presence of multiple cell states within the GBM, with the lineage state representing the cells that actively proliferate and migrate to ensure GBM survival [29, 34]. We show that GBM cells treated with LY341495 show a downregulation of pathways associated with the neural-like, oligodendrocyte-like and astrocyte-like genes, Supplementary Fig. 4g. In addition, quantification of apoptosis/necrosis in the presence of mGluR3 inhibition confirmed no significant increase in apoptotic/necrotic cells, Supplementary Fig. 4h. Taken together, we show that mGluR3 inhibition has no direct cytotoxic impact on GBM cells, however, resulting in crucial transcriptional changes that could be relevant in light of additional cell-stressing influences such as alkylating chemotherapeutics.

### mGluR3 inhibition facilitates TMZ-induced cytotoxicity

Since we present a role of mGluR3 receptor expression and a resistance against alkylating chemotherapeutics, we aimed to overcome chemoresistance by mGluR3 inhibition. Only the combination of mGluR3 inhibition (LY341495) and TMZ showed significant antiproliferative and cytotoxic effects throughout different cell lines (*n* = 3), whereas singular application of TMZ was ineffective. The effect could be potentiated by increasing the dosage of TMZ in the combination therapy from 50 to 100 µM, Fig. 5a, b. In addition, cellular activity was significantly reduced in all three GBM cell lines under the combinatorial treatment with TMZ and LY341495, Fig. 5c, Supplementary Fig. 5a–c. Temporal analysis of apoptosis lead to precisely monitored cytotoxic effects of the combination treatment. Fluorescence imaging revealed that the combination of TMZ (25–50 µM) plus LY341495 (60 nM) was exclusively able to induce cell apoptosis within a short period of time (10–240 min), Fig. 5d. All profiled cell lines showed high sensitivity to the combination treatment, marked by the dramatic increase of early apoptotic pSIVA^+^ cells throughout the treatment. Furthermore, quantifying late apoptotic/dying PI^+^ cells, confirmed the finding that initiation of early apoptosis by TMZ plus mGluR3 inhibition was not just transient, but rather caused definitive cell death in the vast majority of cells, Supplementary Fig. 5d–e. Geneset enrichment analysis of samples treated with TMZ vs TMZ + LY341495 show a suppression of pathways related to nucleosome assembly, DNA packaging complex and nucleosome organization, Fig. 5e.

Due to the fact that in vitro models are limited in recapitulating the complexity of glioblastoma within their neural environment, we made use of our recently established human organotypic section-based GBM model, [11, 12, 29, 35]. We inoculated GBM cells into cultured human cortical organotypic sections. Due to the usage of a known unmethylated TMZ resistant cell line [12], we did not find increased cell death by TMZ treatment alone, but rather increased growth to escape cytotoxicity and sustain clonal growth [36]. The combination of LY341495 and TMZ limited tumor growth within 2 days of treatment, significantly reducing the total tumor area increase over time, Fig. 5f, g. Subsequent immunostaining revealed that the combination treatment resulted in a dramatic alteration of tumor morphology within the GBM model, Fig. 5h.

## Discussion

Glutamatergic signaling is crucial in the maintenance, proliferation and invasive behavior of GBM. Several studies have investigated the capability of excessive paracrine glutamate signaling between neurons and GBM to promote cell proliferation, migration and tumor establishment [23, 24, 37–39]. We present evidence that there is a strong relationship between glutamate release and stressors to the GBM, validated using a spatial multi-omics approach. We found that any alteration of glutamate uptake/release results in significant alteration of GBM behavior, playing an important role in driving proliferation and invasive behavior. Ionotropic and metabotropic receptors play varying but isolated roles, with ionotropic receptors playing a major role in cellular migration/ invasion and metabotropic receptors playing a role in proliferation and therapy resistance. In the healthy brain metabotropic group II receptors have been shown to play an important role in synaptic plasticity, spine maturation and circuit development [30]. There have been several high-profile reports detailing the synaptic integration of GBM into neural circuits to promote tumor proliferation and invasion [1, 2]. Therefore, it would be reasonable to hypothesize that the expression of these receptors might play a similar role in GBM, which arises solely in the neural environment. We explored the transcriptomic distribution of the metabotropic glutamate receptors in the developmental phase of GBM and identified Group II metabotropic receptors as being highly expressed, with mGluR3 being an important modulatory receptor. Our results are in line with previous reports, showing that high GRM3 expression in GBM patients correlates with poorer survival and limited tumor-free survival [40]. Our results further extend the previously reported modulatory role of GRM3 [31, 41], by treatment using a minimally toxic mGluR3 antagonist, LY341495, effective at nanomolar concentrations. Mono treatment with the antagonist had no apparent cytotoxic effects in cell culture. Transcriptional profiling of the GBM cells post treatment detected changes in transcriptional programs, where the cells exhibited a loss of both lineage and reactive transcriptional programs, represented as loss of neuron-like, astrocyte-like and oligodendrocyte-like signatures. In addition, we report a downregulation of pathways that are important in the growth and network formation of GBM. It has been previously shown that GBM cells need this network forming ability to be able to resist chemotherapeutic assault [42]. Therefore, by disrupting this ability to function as a singular entity, it is possible to increase susceptibility to therapy. Our results are in agreement with this hypothesis, where we saw that the efficacy of TMZ as a treatment against GBM was significantly potentiated when coupled with LY341495.

In our work, we provide proof of concept of the idea that subtle changes in the transcriptional programs of the tumor, which might not affect the apparent viability of the GBM, when coupled with standard therapy, could lead to significant enhancement of therapeutic efficacy, even in therapy resistant GBM. By targeting these particular transcriptional features, we show that the GBM cells lose their inherent ability to resist chemotherapeutic agents, leading to increased efficacy even in therapy resistant tumors. In addition, our work demonstrates that some changes in the GBM cannot be recapitulated within cell culture and is better represented within a natural neural environment aptly mimicked by the presented human cortical tissue based GBM model, paving the way towards personalized GBM therapy.

## Supplementary information

Supplementary Material

Supplementary Figure 1

Supplementary Figure 2

Supplementary Figure 3

Supplementary Figure 4

Supplementary Figure 5
