# Supplementary material for: Inhibition of metabotropic glutamate receptor III facilitates sensitization to alkylating chemotherapeutics in glioblastoma
Source: Cell Death Dis. 2021 Jul 21;12(8):723. doi: 10.1038/s41419-021-03937-9 (PMC8295384; doi:10.1038/s41419-021-03937-9)
Supplement: Supplementary file 1 — Supplementary Material [file 41419_2021_3937_MOESM1_ESM.docx]

Supplementary Material:

**Supp. Table 1:**

| **Antibody** | **Type** | **Species** | **Dilution** | **Company** |
| --- | --- | --- | --- | --- |
| mGluR3  Nestin  Alexa Fluor 647  Alexa Fluor 568 | Primary  Primary  Secondary  Secondary | Rabbit  Goat  anti-goat  anti-rabbit | 1:1000  1:1000  1:1000  1:1000 | Abcam, Cambridge, UK  Santa Cruz, Santa Cruz, USA  Thermo Fisher Scientific Inc.  Thermo Fisher Scientific Inc. |

*Supp. Table 1:* Primary and secondary antibodies for immunostaining of glioblastoma cell lines

**Supp. Table 2:**

| **Antibody** | **Type** | **Species** | **Dilution** | **Company** |
| --- | --- | --- | --- | --- |
| Active Caspase 3  Collagen IV  Ki67  Alexa Fluor 647  Alexa Fluor 555  Alexa Fluor 405 | Primary  Primary  Primary  Secondary  Secondary  Secondary | Rabbit  Goat  Mouse  anti-goat  anti-mouse  anti-rabbit | 1:500  1:1000  1:1000  1:1000  1:1000  1:1000 | BD Pharmingen  Agilent Technologies Inc.  DAKO  Thermo Fisher Scientific Inc.  Thermo Fisher Scientific Inc.  Thermo Fisher Scientific Inc. |

*Supp. Table 2:* Primary and secondary antibodies for immunostaining of human organotypic brain sections

**Supp. Table 3:**

| Buffer or Solution | Ingredient | Conc. / Amount | |
| --- | --- | --- | --- |
| Preparation Medium              Growth Medium | Hibernate-A Medium  D-Glucose  N-Methyl-D-Glucamine  GlutaMAX    Neurobasal L-Glutamine  Serum-free B-27  Antibiotic-Antimycotic (100X)  D-Glucose  MgSO4  Hepes  GlutaMAX | 50 ml  13 mM  30 mM  1 mM    50 ml  2%  2%  13 mM  1 mM  15 mM  2 mM |  |

*Supp. Table 3: Preparation Medium for Human Organotypic Slice Model*

Supplementary Figure 1: (a) Intracellular concentration of glutamate from 3 profiled primary GBM cell lines (b) Cell death following treatment with 500μM Sulfasalazine (c) Increase in apoptotic cells was dramatic in SAS treated cells over 30 hours post treatment.

Supplementary Figure 2: Glutamate stimulation curve shows a significant increase of cellular proliferation on glutamate stimulation (b) Expression of glutamatergic receptor genes along the developmental-reactive subtype axis of GBM samples from a publicly available dataset.

Supplementary Figure 3: (a) mRNA expression shows an increased presence of GRM3 positive cells at the leading edge of the tumor, similar to findings from patient data.

Supplementary figure 4: BrdU based proliferation assay post treatment with LY341495 (100nM) (b) Representative images from cell cultures treated with LY341495 shows no change in cell numbers post treatment for 48h (c) Workflow showing the acquisition of cellular kinetic data (d) Velocity heatmaps showing no changes in cellular velocity due to the treatment (e) Histograms of all features studied shows no differences between conditions. (f) Quantification of the total distance travelled by the cells in 72 hours show no difference (p=0.39) (g) GSEA analysis shows significant downregulation of GBM phenotypes post treatment: NPC-like (p. adj=0.0005), OPC-like (p. adj=0.0002) and APC-like (p. adj=0.0000001

Supplementary Figure 5: (a) Velocity heatmaps of cells in all conditions. There is a significant reduction in cellular velocity due to the combinatorial treatment (b) Quantification of the average cellular movement shows a significant reduction of cellular movement due to the combined treatment (p<0.00001) (c) Other features pertaining to cellular kinetics such as maximum speed of the cell and the total distance travelled were significantly altered due to the combinatorial treatment (d) Kinetic apoptosis assay revealed a significant increase of cells in both early and late apoptosis, over 5hours of treatment (e) Representative images of cells that were treated with the combination treatment. Cells that were treated with TMZ alone were minimally affected in comparison to the combinatorial treatment.
