# Supplementary figures and images for: Inhibition of metabotropic glutamate receptor III facilitates sensitization to alkylating chemotherapeutics in glioblastoma
Source: Cell Death Dis. 2021 Jul 21;12(8):723. doi: 10.1038/s41419-021-03937-9 (PMC8295384; doi:10.1038/s41419-021-03937-9)

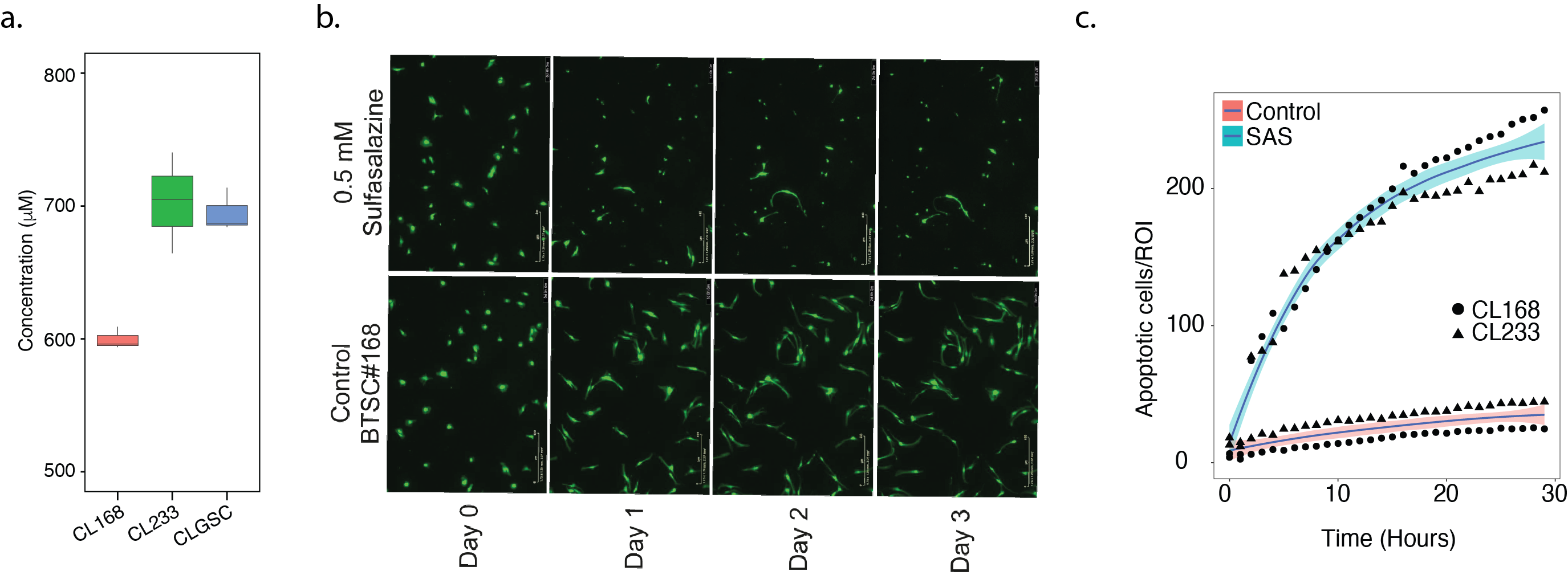

Supplement: Supplementary file 2 — Supplementary Figure 1 [file 41419_2021_3937_MOESM2_ESM.png]

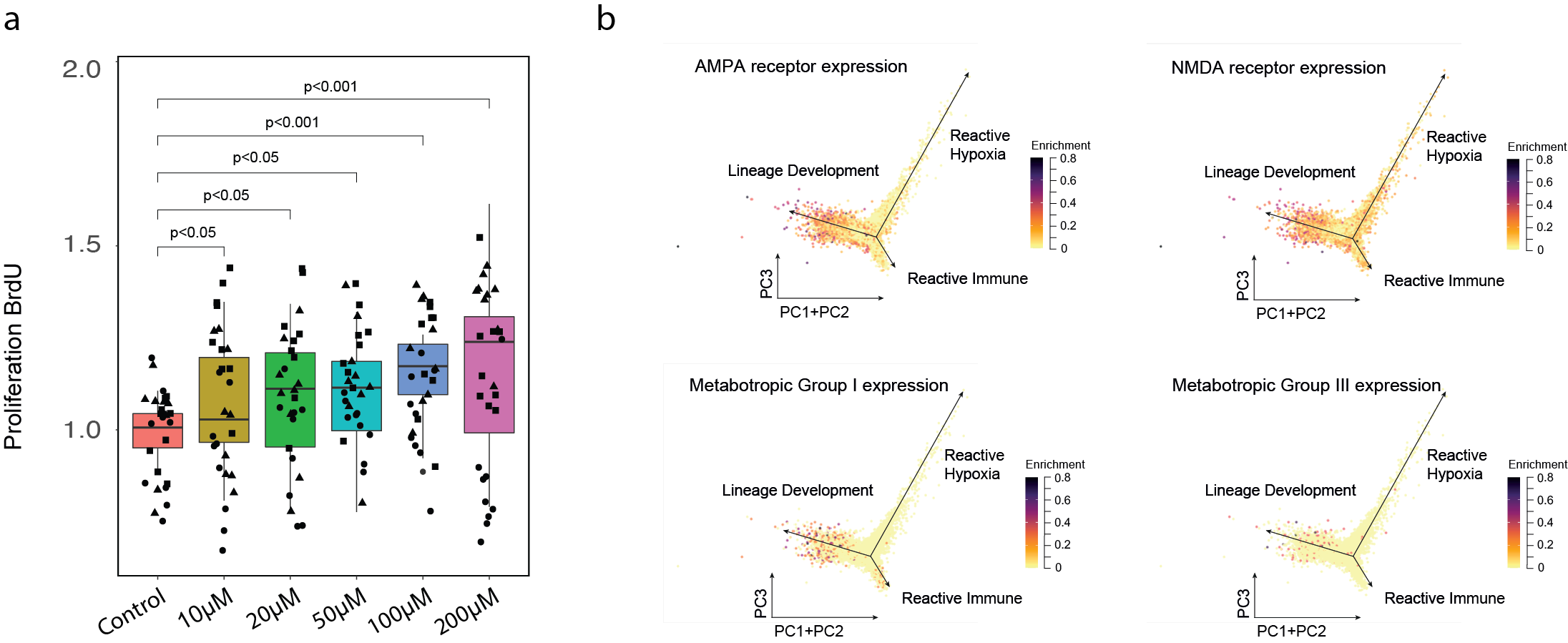

Supplement: Supplementary file 3 — Supplementary Figure 2 [file 41419_2021_3937_MOESM3_ESM.png]

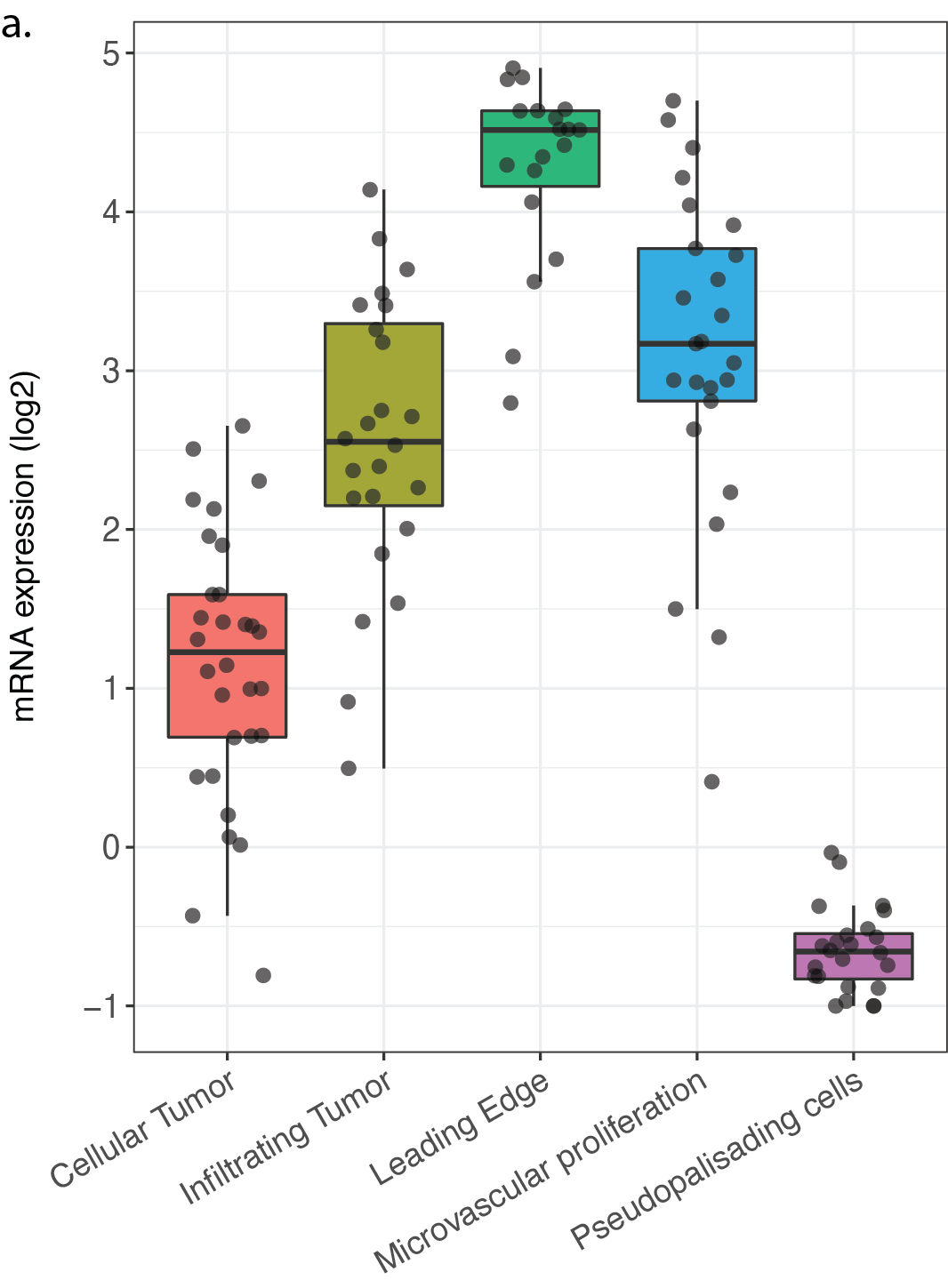

Supplement: Supplementary file 4 — Supplementary Figure 3 [file 41419_2021_3937_MOESM4_ESM.png]

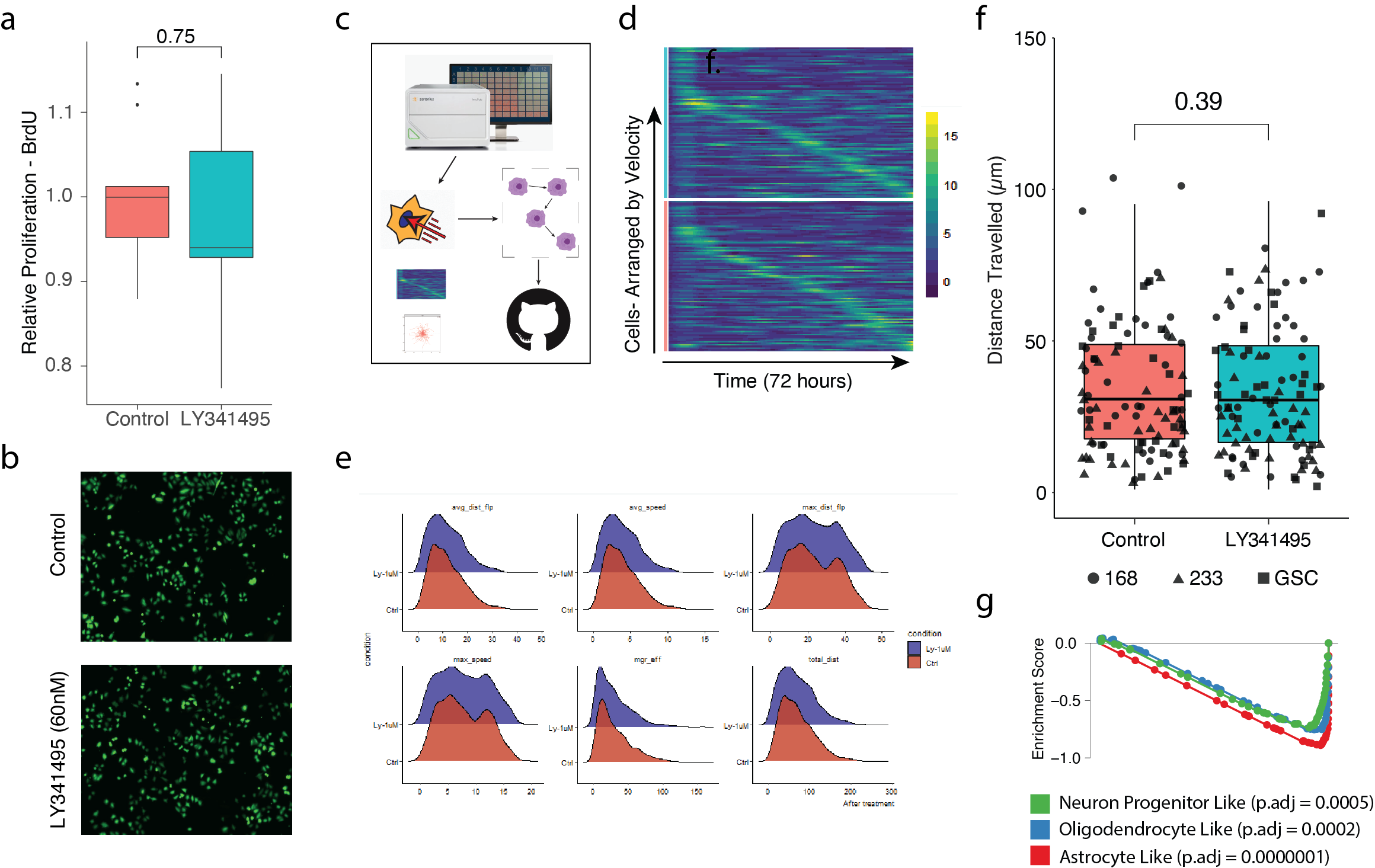

Supplement: Supplementary file 5 — Supplementary Figure 4 [file 41419_2021_3937_MOESM5_ESM.png]

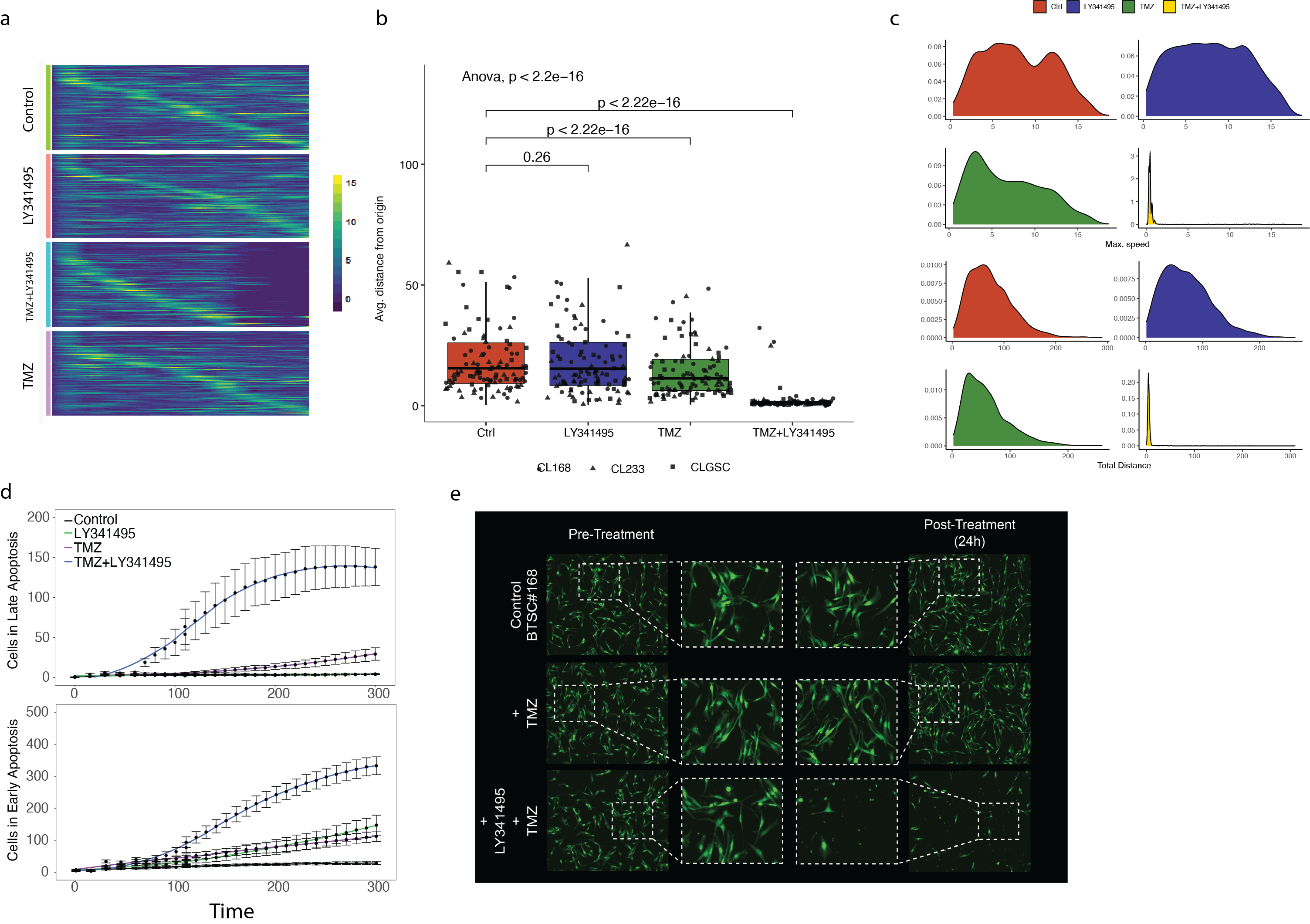

Supplement: Supplementary file 6 — Supplementary Figure 5 [file 41419_2021_3937_MOESM6_ESM.png]
